# Dihydromyricetin alleviates *Escherichia coli* lipopolysaccharide-induced hepatic injury in chickens by inhibiting the NLRP3 inflammasome

**DOI:** 10.1186/s13567-022-01024-1

**Published:** 2022-01-24

**Authors:** Chenxi Shi, Jiaqi Wang, Ruichen Zhang, Muhammad Ishfaq, Ying Li, Ruihui Zhang, Chuanbiao Si, Rui Li, Changwen Li, Fangping Liu

**Affiliations:** 1 Basic Veterinary Department, College of Veterinary Medicine, Northeast Agricultural University, Harbin, China; 2 Heilongjiang Key Laboratory for Animal Disease Control and Pharmaceutical Development, Harbin, China; 3 Laboratory Animal Base, Harbin Veterinary Research Institute of Chinese Academy of Agricultural Sciences, Harbin, China

**Keywords:** Dihydromyricetin, NLRP3 inflammasome, hepatic injury, *Escherichia coli* lipopolysaccharide

## Abstract

Dihydromyricetin (DHM), a flavonoid in vine tea, has many pharmacological activities, including anti-inflammatory and antibacterial effects. Lipopolysaccharide is the key inducer of inflammation in avian pathogenic *Escherichia coli* (*E. coli*) infection; however, the effect of DHM on *E. coli* lipopolysaccharide-induced hepatic injury remains unknown. The present study aimed to explore the role of the NLRP3 inflammasome in hepatic injury and the possible protective mechanisms of DHM against hepatic injury in chickens. The results showed that when chickens were administered lipopolysaccharide, liver damage was observed, accompanied by increased levels of serum transaminases and direct bilirubin. Additionally, hepatic expression levels of NLRP3 and caspase-1 p20, the subunit of caspase-1 that is cleaved after NLRP3 activation, significantly increased in liver injury. We found that treatment with MCC950, a specific NLRP3 inhibitor, significantly decreased serum transaminase activities, direct bilirubin content, and hepatic NLRP3 and caspase-1 p20 expression levels. DHM significantly reduced serum transaminase activities and direct bilirubin content and ameliorated histopathological and ultrastructural changes in the liver. DHM decreased hepatic levels of H_2_O_2_ and malondialdehyde and increased the activities of superoxide dismutase and glutathione peroxidase. Furthermore, DHM significantly decreased the expression levels of NLRP3, pro-caspase-1 and caspase-1 p20. Moreover, DHM reduced serum lactate dehydrogenase, IL-1β and IL-18 levels and repressed hepatic IL-1β, IL-18 and gasdermin A expression. The results demonstrated that the NLRP3 inflammasome was involved in the mechanism of lipopolysaccharide-induced hepatic injury. Furthermore, DHM could inhibit NLRP3 inflammasome activation and subsequent pyroptosis, eventually ameliorating *E. coli* lipopolysaccharide-induced liver injury.

## Introduction

Avian pathogenic *Escherichia coli* (APEC), a gram-negative bacterium, is an important pathogen that causes infectious bacterial diseases, seriously threatening the development of the poultry industry. APEC causes systemic or local infections in chickens, such as enteritis, polyserositis, septicaemia and hepatitis, leading to extensive mortality and very large economic losses to poultry flocks [1]. Chickens are susceptible to APEC, which contributes to the spread of infectious diseases. Lipopolysaccharide (LPS), an important component of gram-negative bacteria, is the key inducer of inflammation in APEC infection [2]; large amounts of LPS are released after bacterial death and enter the liver via the portal vein along with other substances from the intestines. LPS is mainly cleared from blood by the liver; however, the liver can be severely damaged by large doses of LPS [3], which in turn represses the capacity of the liver to clear LPS [4]. Hepatic injury is considered a risk factor for gram-negative bacterial infection because of the inability of the liver to clear bacteria or LPS [5]. Thus, it is necessary to find novel agents that prevent and treat LPS-induced hepatic injury.

The nucleotide-binding and oligomerization domain (NOD)-like receptor pyrin domain-containing 3 (NLRP3) inflammasome is a group of high-molecular-weight cytosolic protein complexes. MCC950 is a specific and potent inhibitor of NLRP3 that directly targets its ATP-hydrolysis motif [6]. Conformational activation of the NLRP3 inflammasome promotes the self-cleavage and activation of pro-caspase-1 (a constituent of the NLRP3 inflammasome). Subsequently, active caspase-1 promotes the cleavage of pro-IL-1β and pro-IL-18 into mature IL-1β and IL-18 [7, 8]. At the same time, active caspase-1 promotes the cleavage of gasdermin protein followed by the formation of pores in the cell membrane [9]. Consequently, the release of proinflammatory cytokines results in a proinflammatory form of cell death called pyroptosis [10, 11]. Pyroptosis is a novel form of programmed cell death and is executed by gasdermins, a family of pore-forming effector proteins. Currently, in chickens, the gasdermin family is considered to be composed of three paralogous genes: gasdermin A, deafness autosomal recessive 59 (DFNB59), and gasdermin E (also known as DFNA5). The NLRP3 inflammasome and pyroptosis have been studied in mammals, but it is unclear whether they are involved in LPS-induced hepatic injury in chickens.

Vine tea, the tender stems and leaves of *Ampelopsis grossedentata* [Hand-Mazz] W.T. Wang is a traditional Chinese herbal plant that has been used for the prevention and treatment of fatty liver, hypertension, and cardiovascular disease for hundreds of years [12]. Dihydromyricetin (DHM), the most abundant flavonoid in vine tea, has many pharmacological activities. Studies have shown that flavonoids do not exert toxic effects, and they are considered nontoxic food supplements [13–15]. Modern pharmacological research has demonstrated that DHM has cardioprotective effects against myocardial ischaemia–reperfusion injury, which were associated with the inhibition of apoptosis [16]. Our previous study demonstrated that DHM attenuates *E. coli* LPS-induced ileum injury and depresses the inflammatory response in chickens [17]. However, little is known about the potential effect of DHM on LPS-induced hepatic injury.

In the present study, the involvement of the NLRP3 inflammasome during LPS-induced hepatic injury was investigated. Furthermore, DHM was used as a feed additive to evaluate the protective effects of DHM against *E. coli* LPS-induced hepatic injury in chickens and to explore the possible mechanisms of the hepatoprotective effects of DHM, focusing on oxidative stress, the NLRP3 inflammasome and pyroptosis, with the goal of providing potential therapeutic strategies for *E. coli* infection and hepatic injury.

## Materials and methods

### Reagents

Commercial kits for the measurements of alanine aminotransferase (ALT, CAT. No. 23501000), aspartate aminotransferase (AST, CAT. No. 23501010), lactate dehydrogenase (LDH, CAT. No. 23502140) and direct bilirubin (DBILI, CAT. No. 23501270) were obtained from SINNOWA Medical Technology Co. Ltd. (Nanjing, China). Anti-NLRP3 (bs-10021R, 1:500), anti-gasdermin A (bs-16331R, 1:500) and anti-caspase1-p20 (bs-10743R, 1:500) antibodies were purchased from Bioss Biotech Co. Ltd. (Beijing, China). LPS (O55:B5, *E. coli*, CAT. No. L2880) was obtained from Sigma–Aldrich (St. Louis, MO, USA). DHM (extracted from vine tea, purity ≥ 98.0%, detected by high-performance liquid chromatography, CAS No. 27200-12-0) was obtained from Shanghai Winherb Medical Technology Co. Ltd. (Shanghai, China). Commercial kits for the measurements of superoxide dismutase (SOD, CAT. No. A001-1-2), malondialdehyde (MDA, CAT. No. A003-1-2), glutathione peroxidase (GSH-Px, CAT. No. A005-1-2) and H_2_O_2_ (CAT. No. A064-1-1) were purchased from Nanjing Jiancheng Institute of Biotechnology (Nanjing, China). The potent and selective NLRP3 inhibitor MCC950 (CAT. No. S7809) was purchased from Selleck Chemicals (Houston, TX, USA). ELISA kits for chicken IL-1β (CAT. No. CK-E60017) and IL-18 (CAT. No. CK-E60007) were purchased from Kenuodi (Quanzhou, China).

### Animal grouping and treatment

One-day-old Hy-line variety white broiler chicks were purchased from Xianfeng Chicken Farm (Harbin, China). The chickens were fed for 1 week until they were 7 days old to adapt to experimental conditions prior to experiments. Chickens were housed under constant temperature (22 ± 2 ℃), relative humidity (50–60%), and light (12 h light–dark cycles) conditions with a standard laboratory diet and fresh drinking water provided ad libitum. All procedures involving animals complied with the Guiding Principles in the Use of Animals in Toxicology and the National Institutes of Health Guide for the Care and Use of Laboratory Animals. The protocol was approved by the Institutional Animal Care and Use Committee of Northeast Agricultural University (Permit Number: NEAUEC20200317). Experiments were carried out as follows. (1) To assess whether the NLRP3 inflammasome is involved in LPS-induced hepatic injury, 72 fasted chickens (36 male and 36 female) were randomly divided into four groups each with three replicates: the control group, the LPS group (60 mg/kg), the LPS plus MCC950 group (60 mg/kg LPS + 50 mg/kg MCC950), and the MCC950 group (50 mg/kg). Chickens in the LPS + MCC950 group and the MCC950 group received an intraperitoneal injection of MCC950, which was dissolved in saline. Chickens in the control group and the LPS group received an injection of the same amount of saline. Two hours later, the chickens were given an intraperitoneal injection of LPS or saline. The LPS dose used was based on our preliminary experiments, and the MCC950 dose was selected according to published studies [18–20]. (2) To evaluate the effects of DHM on LPS-induced hepatic injury, 108 chickens (54 male and 54 female) were randomly divided into six groups each with three replicates: the control group, the LPS group (60 mg/kg), the LPS (60 mg/kg) plus 0.025% DHM group, the LPS (60 mg/kg) plus 0.05% DHM group, the LPS (60 mg/kg) plus 0.1% DHM group, and the 0.1% DHM group. Chickens in the DHM group were fed a diet mixed with 0.025%, 0.05% or 0.1% DHM (mass fraction) for 14 days. The dose of DHM was chosen according to our previous experiment [17]. The chickens in the control group and LPS group were fed a normal diet. Then, the chickens were given LPS or saline by intraperitoneal injection. Twelve hours after the injection of LPS, the chickens were humanely sacrificed, and blood and the liver were collected for further experimental analyses.

### RNA extraction and quantitative real-time polymerase chain reaction

Total RNA was extracted by TRIzol reagent (CAT. No. 9101, Takara, Dalian, China), and a NanoDrop (Thermo Fisher) was used to evaluate the purity and quantity of RNA by spectrophotometric analysis at OD260/280. Total RNA was reverse-transcribed to cDNA using the PrimeScript™ RT reagent kit with gDNA Eraser (CAT. No. RR047A, Takara, Dalian, China) following the manufacturer’s instructions. Quantitative real-time polymerase chain reaction (q-RT PCR) was performed using a TB Green® Premix Ex Taq™ II (Tli RNaseH Plus) kit (CAT. No. RR820A, Takara, Dalian, China) in a Roche LightCycle® 96 instrument (Shanghai, China) following the manufacturer’s instructions. The qRT–PCR system consisted of 10 μL TB Green Premix Ex Taq II (Tli RNaseH Plus), 2 μL cDNA, 1 μL forward primer (20 mM), 1 μL reverse primer (20 mM), and 6 μL nuclease-free water. All the primers are shown in Table 1. The 2^−ΔΔCt^ method was used to calculate the changes in gene expression levels [21], and the relative mRNA expression levels of genes were normalized to the mRNA expression level of the endogenous control β-actin.

**Table 1:** **Genes and primers used in this study**

Accession No.	Gene names	Sequence (5′ to 3′)		Product
NM_205518.1	β-actin	Forward	CTCTGACTGACCGCGTTACT	172 bp
		Reverse	TACCAACCATCACACCCTGAT	
XM_025142104.1	Pro-caspase-1	Forward	GTGCTGCCGTGGAGACAACATAG	179 bp
		Reverse	AGGAGACAGTATCAGGCGTGGAAG	
NM_001348947.1	NLRP3	Forward	GCTCCTTGCGTGCTCTAAGACC	150 bp
		Reverse	TTGTGCTTCCAGATGCCGTCAG	
NM_204524.1	IL-1β	Forward	AGCAGCCTCAGCGAAGAGACC	90 bp
		Reverse	GTCCACTGTGGTGTGCTCAGAATC	
XM_426573.6	DFNB59	Forward	ACCAAACACGAGGTTGAAGTACCG	121 bp
		Reverse	TGACAACGCACAGAATCTCCATCC	
NM_001031361.1	gasdermin A	Forward	AGCCTCACAGAAGCCATCTCCTAC	196 bp
		Reverse	GCTGCTGCTGCTCGCTGAAG	
NM_001006361.1	gasdermin E	Forward	GCTGCGTGCCTGCTCTGATC	88 bp
		Reverse	GCTCAGTGCCAAGGTGCCATC	
NM_204608.1	IL-18	Forward	AGATGATGAGCTGGAATGCGATGC	97 bp
		Reverse	ATCTGGACGAACCACAAGCAACTG	

### Enzyme-linked immunosorbent assay (ELISA)

Levels of chicken IL-1β and IL-18 in serum were measured with chicken IL-1β and IL-18 ELISA kits according to the manufacturer’s instructions.

### Measurement of biochemical parameters

Levels of chicken ALT, AST, LDH and DBILI in serum were measured by a biochemistry analyser (SINNOWA, Nanjing, China).

### Measurement of oxidative stress

Levels of SOD, MDA, GSH-Px and H_2_O_2_ in the liver were detected by commercial kits according to the manufacturer’s instructions.

### Western blot analysis

The expression levels of NLRP3, gasdermin A and caspase1-p20 were detected by Western blot analysis according to a previously described method [22]. Briefly, total proteins were extracted from liver tissues using a total protein extraction kit (CAT. No. P0013, Beyotime Biotechnology Co. Ltd, Shanghai, China). A bicinchoninic acid protein assay kit (CAT. No. P0010, Beyotime Biotechnology Co. Ltd, Shanghai, China) was used to measure the protein concentrations. The samples (40 μg protein/lane) were separated by electrophoresis using 10–15% sodium dodecyl sulfate–polyacrylamide gel electrophoresis, and proteins were transferred to nitrocellulose membranes. The membranes were incubated with primary antibodies at 4 °C for 12 h and HRP-conjugated secondary antibody at 37 °C for 2 h. The immunopositive bands were detected by the enhanced chemiluminescence method using a chemiluminescence system (Affinity Biosciences, Ohio, USA). Densitometric analysis of the target bands was performed with ImageJ (National Center for Biotechnology Information, NCBI), and β-actin expression was used as an endogenous control for protein expression analysis.

### Histopathological analyses

Liver tissues were fixed in 10% neutral phosphate-buffered formalin, dehydrated in a graded ethanol series ranging from 50 to 100%, cleared in xylene, and embedded in paraffin. Five-micrometre-thick sections were prepared, stained with haematoxylin and eosin dye (H&E), and imaged with an optical microscope (Nikon E100, Tokyo, Japan).

### Transmission electron microscope observation

Ultrastructural observation was carried out by transmission electron microscopy (TEM). Liver tissues (< 1 mm^3^) were fixed in 2.5% glutaraldehyde for 3 h and rinsed in 0.1 M phosphate buffer for 15 min three times. Then, the samples were fixed in 1% osmium tetroxide for 3 h, rinsed with 0.1 M phosphate buffer for 15 min three times, dehydrated in a graded ethanol and acetone series, and embedded in epoxy resin. Then, the samples were sectioned with an ultramicrotome (EM UC7, Leica, Wetzlar, Germany), stained with uranyl acetate and lead citrate and observed under a transmission electron microscope (GEM-1200ES, JEOL Ltd., Tokyo, Japan).

### Statistical analysis

Statistical analyses were carried out using SPSS version 19.0 software (SPSS, Inc., Chicago, IL, USA). Data were presented as the mean ± standard deviation (SD). The differences among different groups were analysed by one-way analysis of variance (ANOVA). A *P* value < 0.05 was considered statistically significant with Tukey’s multiple comparison test.

## Results

### LPS-induced hepatic injury in chickens

**Figure 1:**
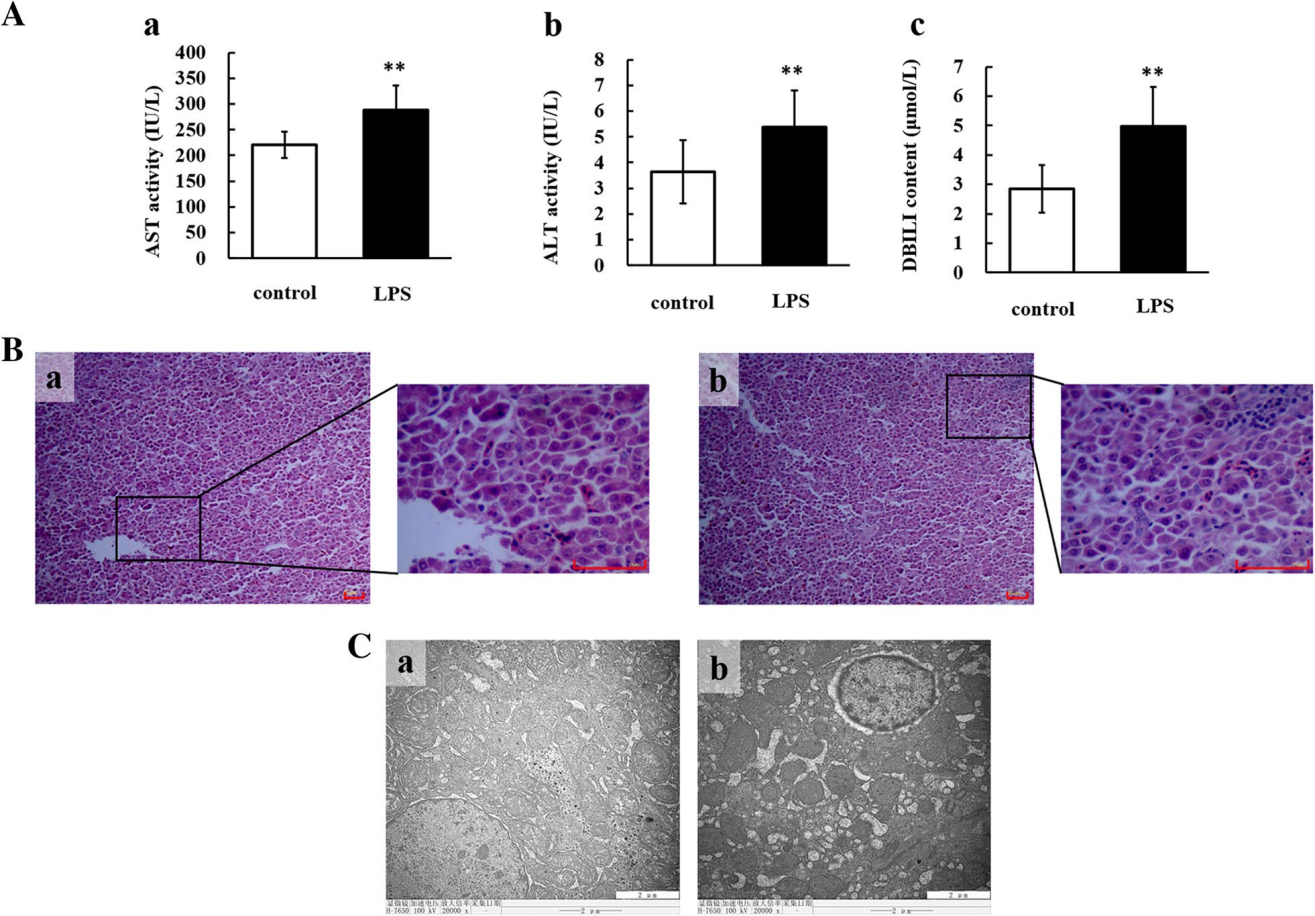
**LPS induced hepatotoxicity in chickens.** **A** Changes in AST activity (**a**), ALT activity (**b**), and DBILI content (**c**) in serum after LPS treatment (60 mg/kg). ** *P* < 0.01 vs. the control group. **B** Histopathological analysis of chicken liver. Histopathological examination results of liver samples from two experimental groups are shown (scale bar = 50 μm), and the images are displayed at 100 × and 400 × the original magnification. The experimental groups were the control group (**a**) and LPS-treated group (**b**). **C** Ultrastructural analysis of chicken liver. Transmission electron microscopic examination of liver samples from two experimental groups (scale bar = 2 μm). **a** shows the control group, and **b** shows the LPS-treated group. ALT: alanine aminotransferase; AST: aspartate aminotransferase; DBILI: direct bilirubin.

Hepatic injury caused by LPS was evaluated by measuring biochemical parameters. As shown in Figure 1A, serum AST and ALT activities and DBILI content significantly increased (*P* < 0.01) in chickens treated with LPS compared with control chickens. Histopathological analysis showed that there were more inflammatory cells and hepatic cell cord fragmentation in the liver in the LPS-treated group than in the control group (Figure 1B). Ultrastructural analysis showed that liver tissues from LPS-treated chickens had chromatin aggregation around the nuclear membrane, increased space between the inner and outer nuclear membrane, decreased glycogen and swollen mitochondria compared with liver tissues from saline-treated chickens (Figure 1C). Taken together, these results show that LPS could cause severe hepatic injury.

### The NLRP3 inflammasome participates in LPS-induced hepatic injury

**Figure 2:**
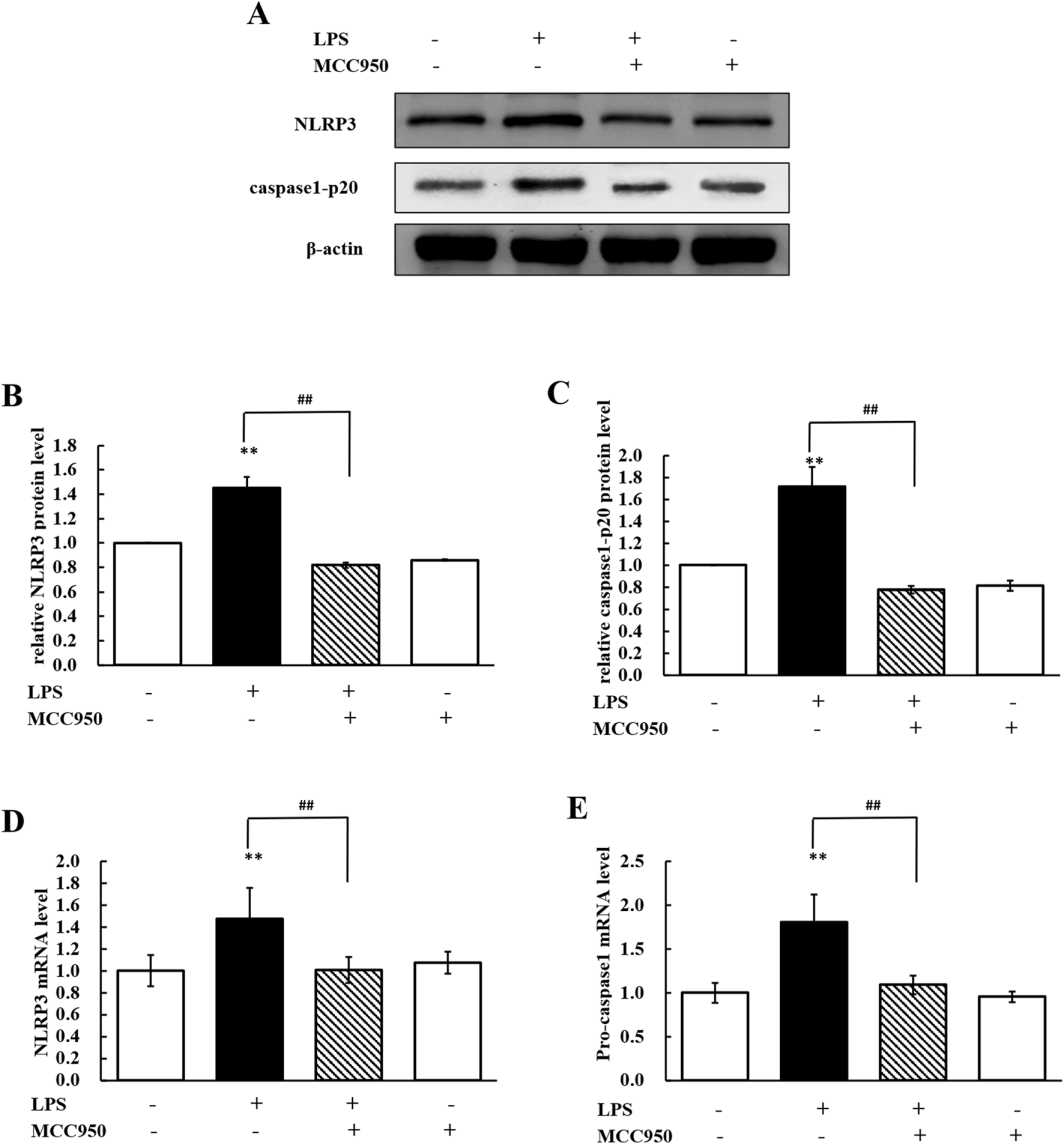
**MCC950 repressed the activation of the NLRP3 inflammasome.** **A** Western blot analyses of the expression of NLRP3, caspase1-p20, and β-actin (loading control) in total tissue lysates. **B**, **C** Changes in the protein expression levels of NLRP3 (fold change in NLRP3/β-actin) and caspase1-p20 (fold change in caspase1-p20/β-actin) in livers from chickens administered MCC950 (5 mg/kg) followed by LPS (60 mg/kg). **D**, **E** Changes in the mRNA expression levels of NLRP3 (fold change in NLRP3/β-actin) and pro-caspase-1 (fold change in pro-caspase-1/β-actin) in livers from chickens stimulated by LPS (60 mg/kg) with or without MCC950 administration (5 mg/kg). Values are expressed as the mean ± SD for each group (*n* = 6). **P < 0.01 vs. the control group. ^##^*P* < 0.01 vs. the LPS-treated group.

The expression of NLRP3 and caspase-1 after LPS administration was detected (Figure 2). The mRNA and protein expression levels of NLRP3 were significantly increased (*P* < 0.01) in the LPS group compared to the control group. The mRNA expression level of pro-caspase-1 significantly increased (*P* < 0.01) after LPS treatment (Figure 2D). Cleaved caspase-1 is often used as a characteristic indicator of NLRP3 inflammasome activation [23]. The protein expression level of caspase-1 p20 was significantly increased (*P* < 0.01) in the LPS group compared with the control group (Figure 2C). The results indicated that the NLRP3 inflammasome was activated in chicken liver after LPS treatment.

To confirm whether the NLRP3 inflammasome participates in LPS-induced hepatic injury, the NLRP3 inhibitor MCC950 was used to block the activation of NLRP3. Lower expression levels of NLRP3 and caspase-1 p20 were observed in chickens cotreated with MCC950 and LPS than in chickens treated with LPS (Figures 2A, B and C). As shown in Figures 2D and E, compared with LPS treatment alone, MCC950 and LPS cotreatment significantly reduced (*P* < 0.01) the mRNA expression levels of NLRP3 and caspase-1. The results demonstrated that the NLRP3 inflammasome was activated in the liver after chickens were treated with LPS and that MCC950 inhibited the activation of NLRP3.

**Figure 3:**
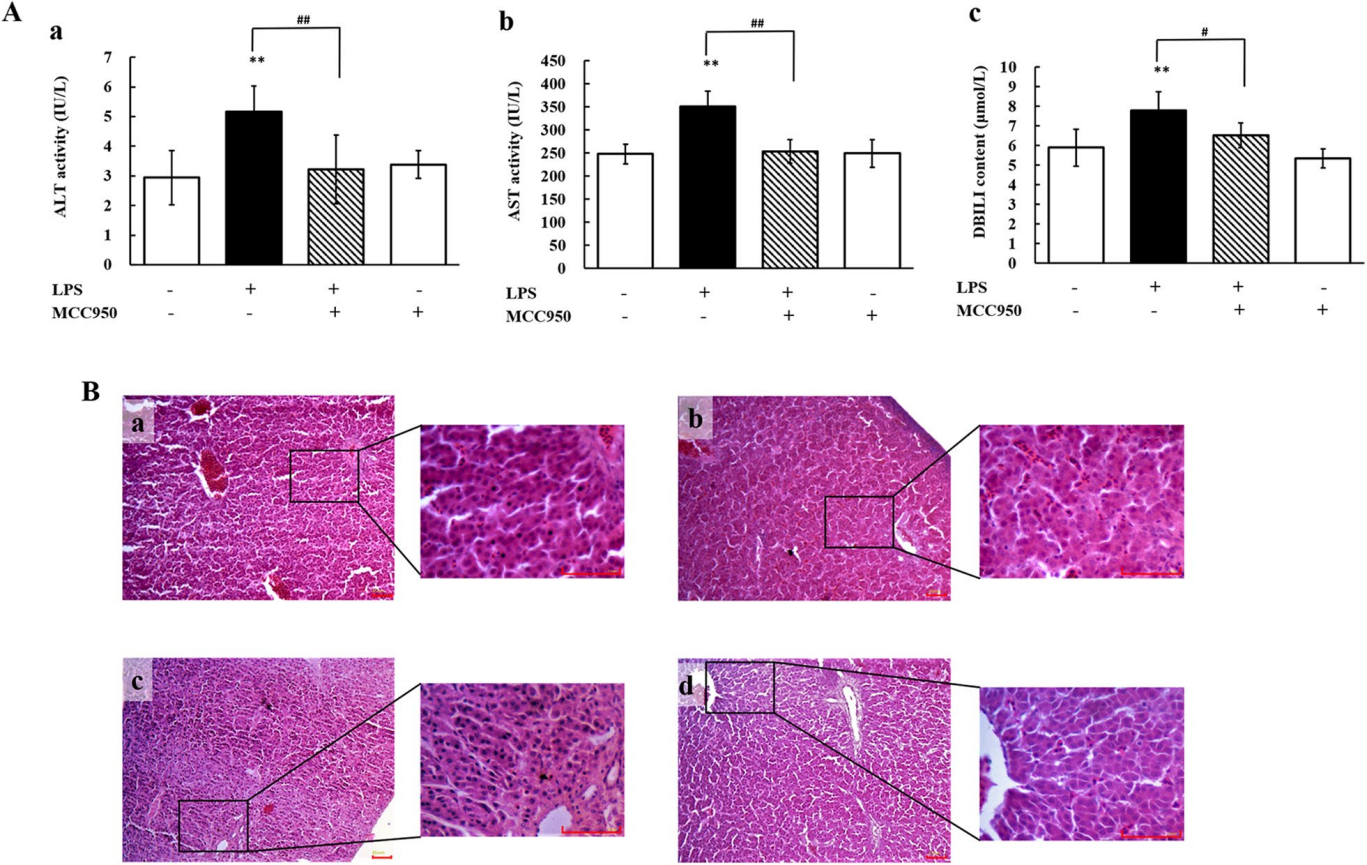
**NLRP3 participates in LPS-induced hepatotoxicity.** **A** Changes in AST activity (**a**), ALT activity (**b**), and DBILI content (**c**) in serum from chickens administered MCC950 (5 mg/kg) followed by exposure to LPS (60 mg/kg). Values are expressed as the mean ± SD for each group (*n* = 6). **P < 0.01 vs. the control group. ^##^*P* < 0.01 vs. the LPS-treated group. **B** Histopathological analysis of chicken liver. The images are displayed at 100 × and 400 × the original magnification (scale bar = 50 μm). The experimental groups were the control group (**a**), LPS-treated group (**b**), MCC950 + LPS group (**c**), and MCC950 alone group (**d**). ALT: alanine aminotransferase; AST: aspartate aminotransferase; DBILI: direct bilirubin.

We assessed serum levels of ALT, AST and DBILI and performed histopathological analyses of the liver after inhibiting NLRP3. As shown in Figure 3A, serum levels of ALT, AST and DBILI were significantly decreased (*P* < 0.01) in MCC950 + LPS-treated chickens compared with LPS-treated chickens. Liver tissues from chickens cotreated with MCC950 and LPS showed milder hepatocellular degeneration and better liver lobule structure than those from chickens treated with LPS as evidenced by histopathological analyses (Figure 3B). These results revealed that the NLRP3 inflammasome participated in LPS-induced hepatic injury.

### Pyroptosis occurs in LPS-induced hepatic injury

**Figure 4:**
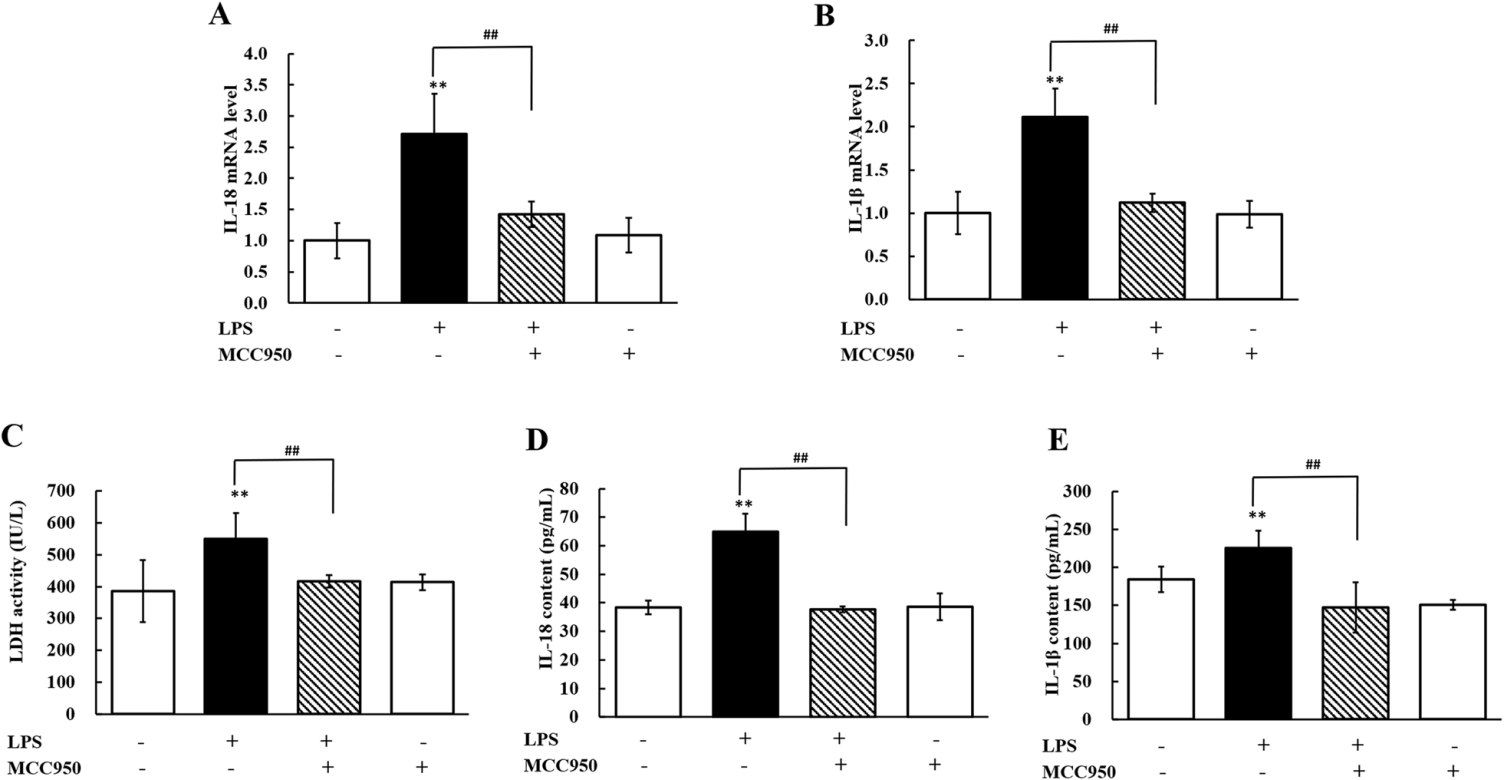
**Pyroptosis occurs in LPS-induced hepatotoxicity.** **A**, **B** Changes in the mRNA expression levels of IL-18 (fold change in IL-18/β-actin) and IL-1β (fold change in IL-1β/β-actin) in livers from chickens stimulated by LPS (60 mg/kg) with or without MCC950 administration (5 mg/kg). **C**–**E** Changes in LDH activity, IL-18 content and IL-1β content in serum from chickens administered MCC950 (5 mg/kg) followed by LPS exposure (60 mg/kg). Values are expressed as the mean ± SD for each group (*n* = 6). **P < 0.01 vs. the control group. ^##^*P* < 0.01 vs. the LPS-treated group. LDH: lactate dehydrogenase.

Compared with the control condition, LPS treatment significantly increased (*P* < 0.01) the mRNA expression levels of IL-1β and IL-18 in the liver (Figures 4A and B), while treatment with MCC950 significantly repressed (*P* < 0.01) their expression levels. The serum LDH, IL-1β and IL-18 levels in the LPS group were significantly increased (*P* < 0.01) compared to those in the control group. When chickens were cotreated with MCC950 and LPS, the contents of serum IL-1β and IL-18 and the activity of LDH significantly decreased (*P* < 0.01) relative to treatment with LPS (Figures 4C, D and E). These data indicated that pyroptosis occurred in hepatic injury caused by LPS and that inhibiting NLRP3 could repress pyroptosis.

### DHM alleviates LPS-induced hepatic injury

**Figure 5:**
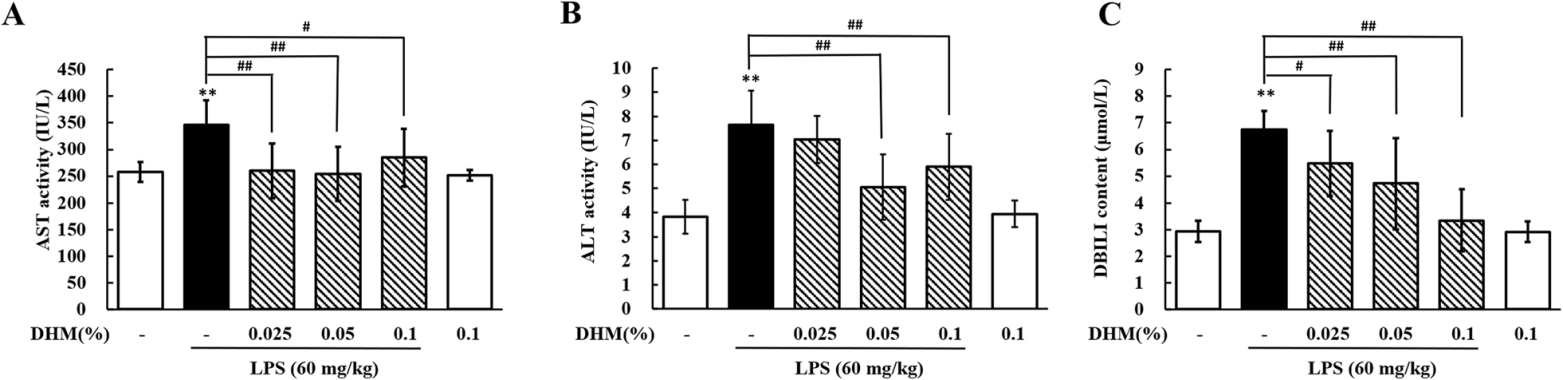
**Protective effects of DHM against LPS-induced hepatotoxicity.** Changes in AST activity (**A**), ALT activity (**B**), and DBILI content (**C**) in serum from chickens administered 0.025%, 0.05% or 0.1% DHM for 14 days followed by LPS exposure (60 mg/kg). Values are expressed as the mean ± SD for each group (*n* = 6). **P < 0.01 vs. the control group. #*P* < 0.05 and ##*P* < 0.01 vs. the LPS-treated group. ALT: alanine aminotransferase; AST: aspartate aminotransferase; DBILI: direct bilirubin.

ALT and AST activities and DBILI content in serum were measured following the administration of DHM, and the results are presented in Figure 5. Compared with LPS treatment, 0.025% DHM plus LPS treatment significantly repressed serum AST activity (*P* < 0.01) and DBILI content (*P* < 0.05), and the 0.05% and 0.1% DHM treatments significantly decreased ALT and AST activities and DBILI content (*P* < 0.01). It is interesting that the 0.1% DHM treatment had no significant effect on these biochemical parameters compared to the control condition.

**Figure 6:**
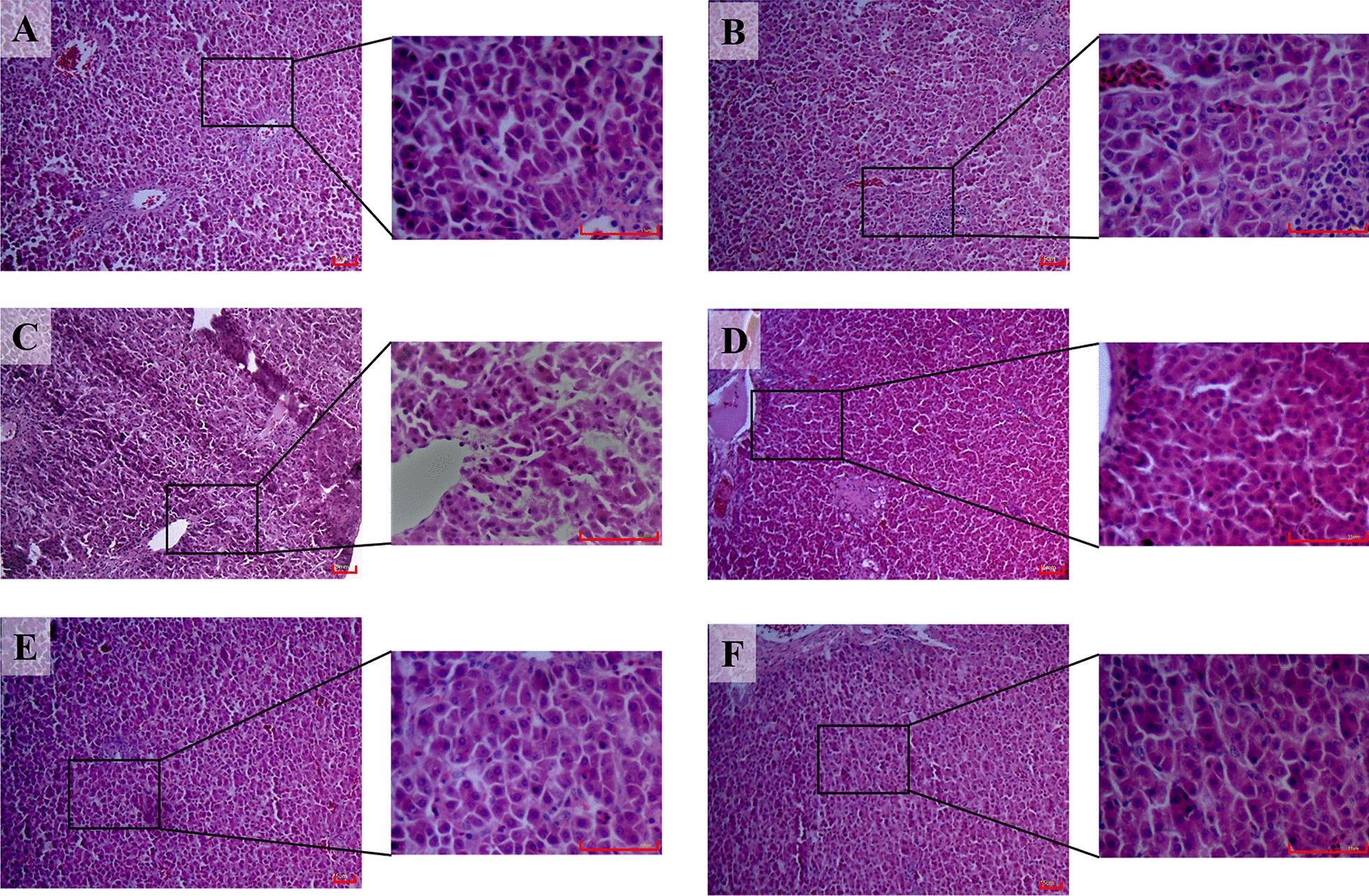
**Histopathological analysis of chicken liver.** Histopathological examination of liver samples from six experimental groups. The images are displayed at 100 × and 400 × the original magnification (scale bar = 50 μm). The experimental groups were the control group (**A**), LPS-treated group (**B**), 0.025% DHM + LPS group (**C**), 0.05% DHM + LPS group (**D**), 0.1% DHM + LPS group (**E**), and 0.1% DHM alone group (**F**).

Histopathological observation showed normal hepatocytes, lobules and central veins in the control group (Figure 6A) and DHM group (Figure 6F). Destroyed hepatic cords, bleeding points and inflammatory cell infiltration were observed in the LPS-treated group (Figure 6B). There were still some inflammatory cells and disordered hepatic cords in the liver from the 0.025% DHM group (Figure 6C). In contrast, 0.05% DHM treatment dramatically reduced inflammatory infiltration (Figure 6D). Tissues from 0.1% DHM-treated chickens showed normal hepatic cords and central veins (Figure 6E).

**Figure 7:**
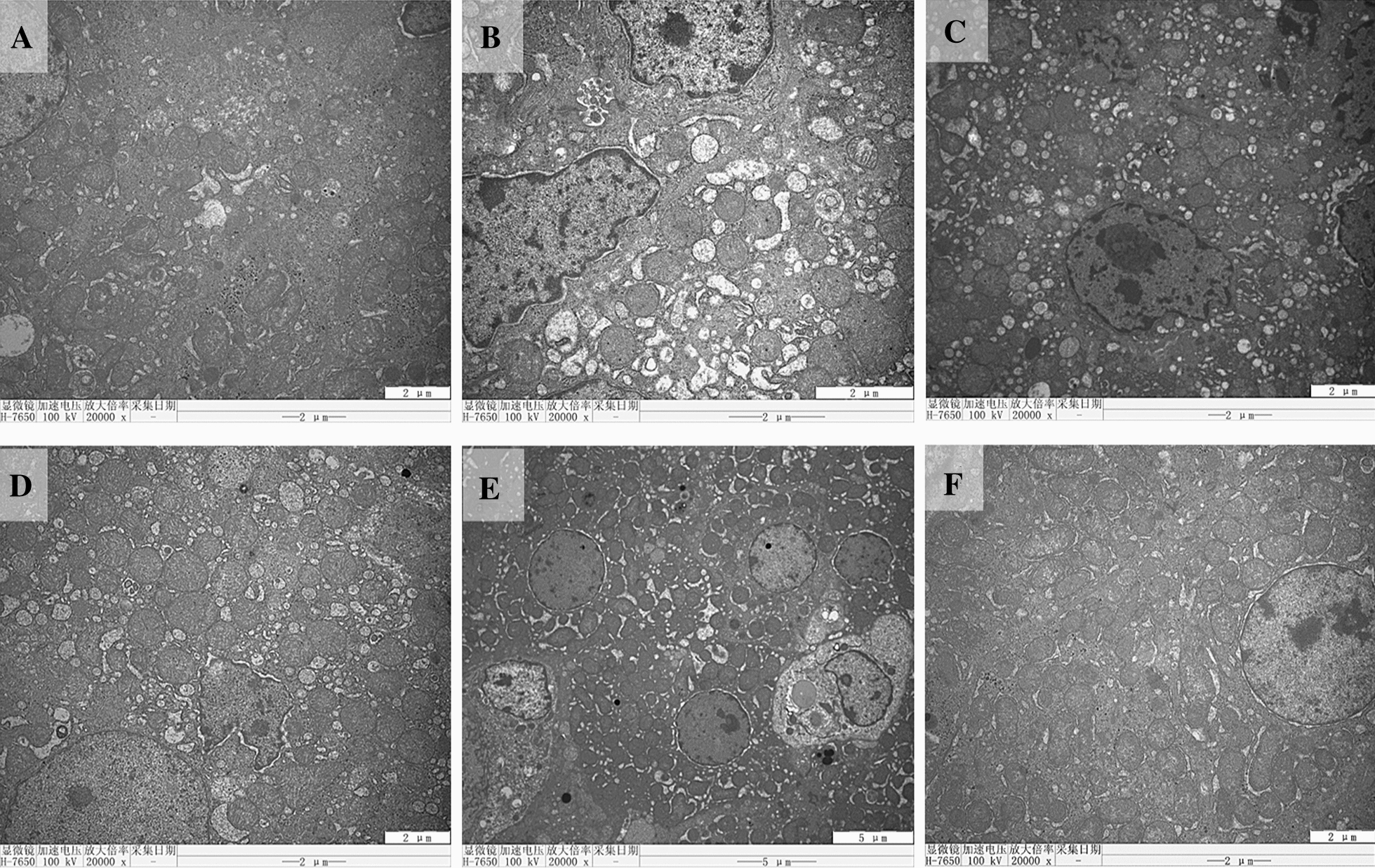
**Ultrastructural analysis of chicken liver.** Transmission electron microscopic images of liver samples from six experimental groups are shown (scale bar = 5 μm). The experimental groups were the control group (**A**), LPS-treated group (**B**), 0.025% DHM + LPS group (**C**), 0.05% DHM + LPS group (**D**), 0.1% DHM + LPS group (**E**), and 0.1% DHM alone group (**F**).

Ultrastructural observation of liver tissues from the control group (Figure 7A) and DHM group (Figure 7F) showed normal nuclei, glycogen, endoplasmic reticulum, and mitochondria with well-arranged cristae. In contrast, liver tissues from the LPS group showed numerous vacuoles, swollen mitochondria with invisible cristae, chromatin aggregation and low glycogen (Figure 7B). Small vacuoles and swollen mitochondria were observed in the 0.025% DHM group (Figure 7C). Moreover, the 0.05% (Figure 7D) and 0.1% (Figure 7E) DHM treatments dramatically reduced the occurrence of vacuoles and swollen mitochondria and increased the level of glycogen and the presence of normal mitochondria. These data indicated that DHM protected against hepatic injury caused by *E. coli* LPS in a dose-dependent manner.

### DHM reduces oxidative stress in LPS-induced hepatic injury

**Figure 8:**
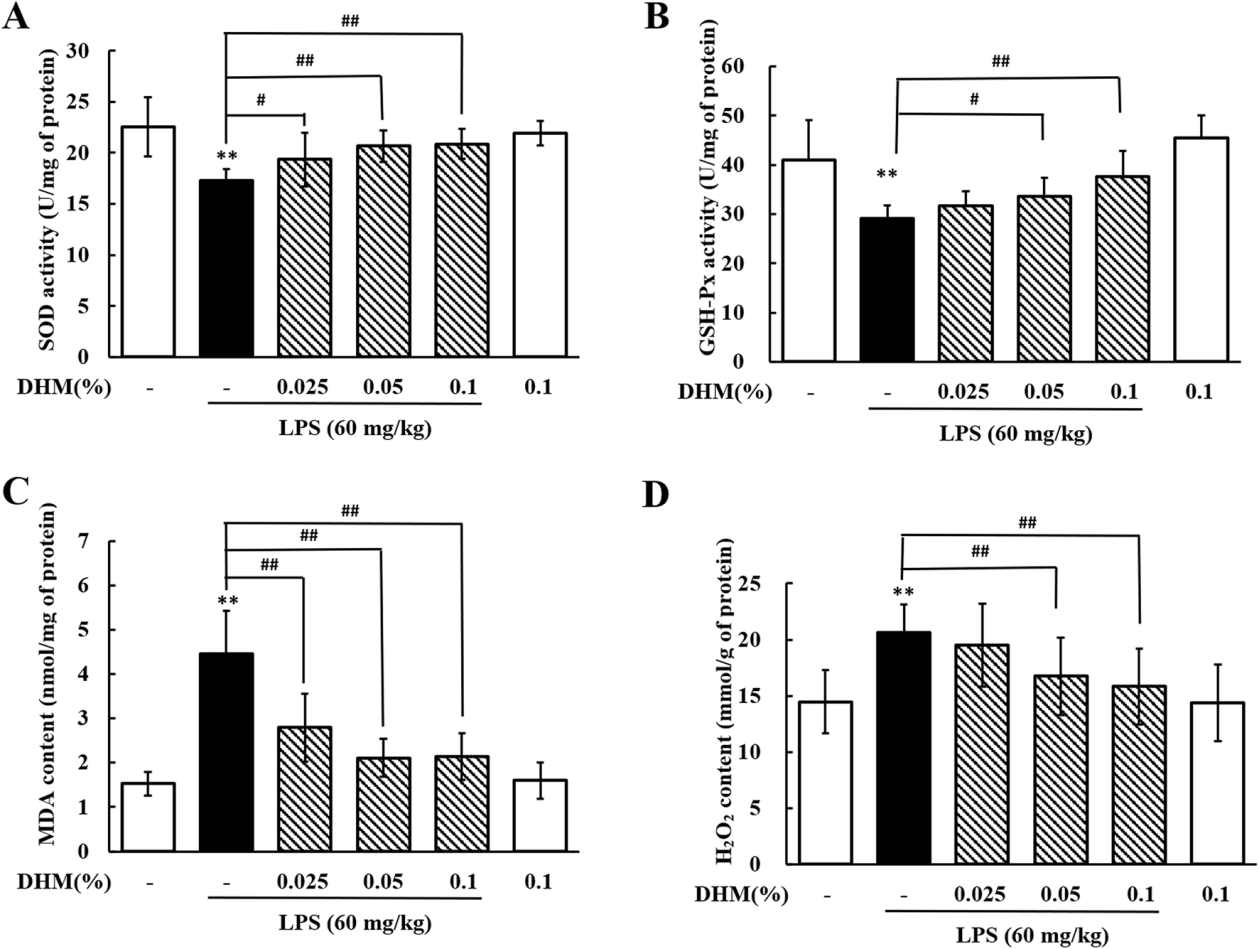
**Effects of DHM on oxidative stress in LPS-induced hepatotoxicity.** Changes in SOD activity (**A**), GSH-Px activity (**B**), MDA content (**C**), and H_2_O_2_ content (**D**) in liver from chickens administered 0.025%, 0.05% or 0.1% DHM for 14 days followed by LPS exposure (60 mg/kg). Values are expressed as the mean ± SD for each group (*n* = 6). **P < 0.01 vs. the control group. ^#^*P* < 0.05 and ^##^*P* < 0.01 vs. the LPS-treated group. SOD: superoxide dismutase; GSH-Px: glutathione peroxidase; MDA: malondialdehyde.

As shown in Figure 8, the liver SOD and GSH-Px activities in the LPS group were significantly decreased compared to those in the control group (*P* < 0.01). Compared with the LPS group, the 0.025% DHM group showed markedly increased SOD activity (*P* < 0.05), the 0.05% DHM group showed significantly increased SOD (*P* < 0.01) and GSH-Px (*P* < 0.05) activities, and the 0.1% DHM group showed significantly increased SOD and GSH-Px activities (*P* < 0.01). LPS treatment significantly increased MDA and H_2_O_2_ contents in the liver (*P* < 0.01). However, compared to LPS treatment, 0.025% DHM treatment significantly decreased (*P* < 0.01) MDA content, and 0.05% and 0.1% DHM treatment significantly decreased (*P* < 0.01) MDA and H_2_O_2_ contents. Interestingly, treatment with DHM alone did not change oxidative stress-related parameters. These data suggested that oxidative stress was promoted by LPS, while DHM had a potent inhibitory effect on oxidative stress.

### DHM represses NLRP3 inflammasome activation in LPS-induced hepatic injury

**Figure 9:**
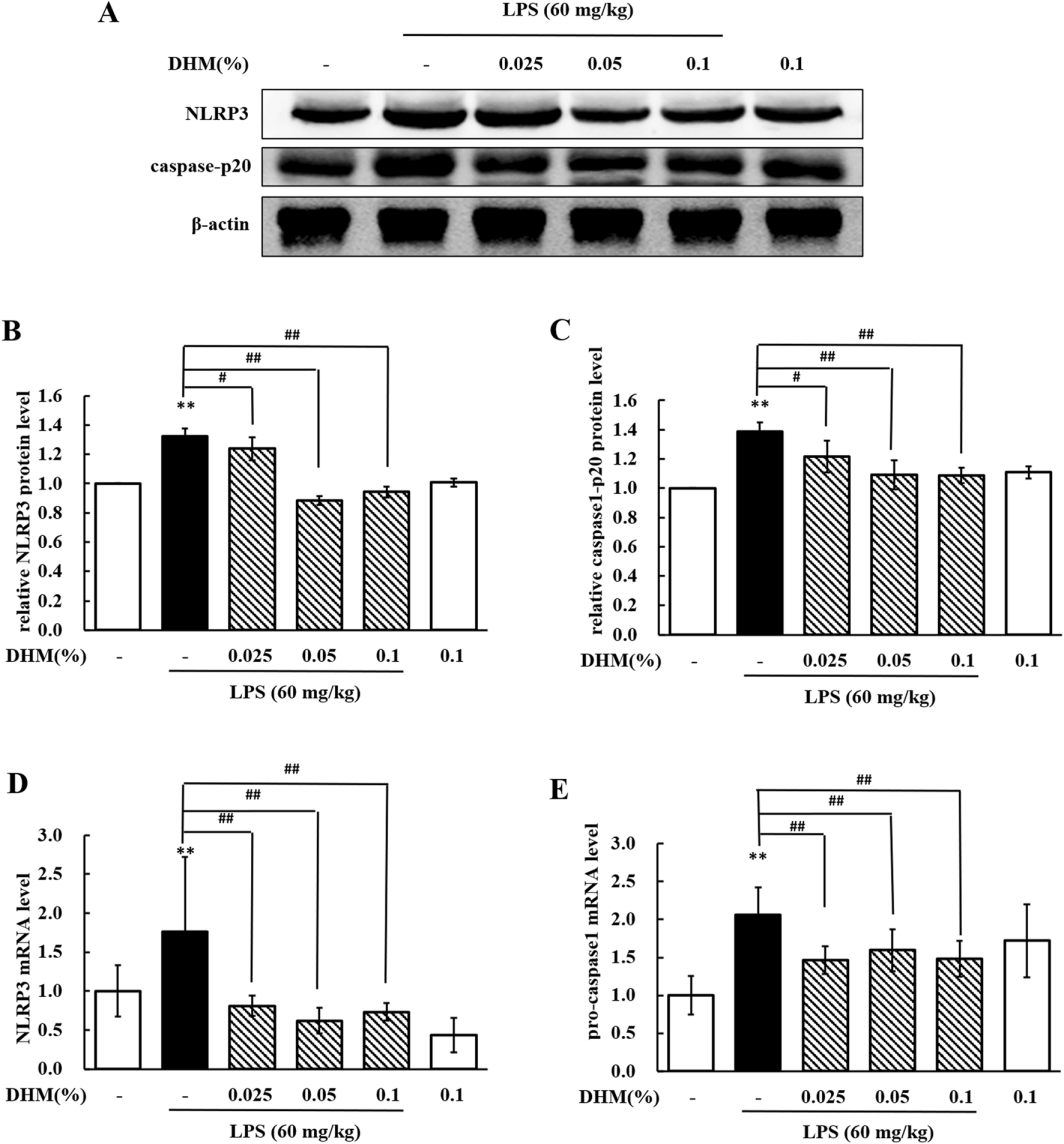
**DHM repressed NLRP3 inflammasome activation in LPS-induced hepatotoxicity**. **A** Western blot analyses of NLRP3, caspase1-p20, and β-actin (loading control) expression in total tissue lysates. **B**, **C** Changes in the protein expression levels of NLRP3 (fold change in NLRP3/β-actin) and caspase1-p20 (fold change in caspase1-p20/β-actin) in livers from chickens administered 0.025%, 0.05% or 0.1% DHM for 14 days followed by LPS exposure (60 mg/kg). **D**, **E** Changes in the mRNA expression levels of NLRP3 (fold change in NLRP3/β-actin) and pro-caspase-1 (fold change in pro-caspase-1/β-actin) in livers from chickens administered 0.025%, 0.05% or 0.1% DHM for 14 days followed by LPS exposure (60 mg/kg). Values are expressed as the mean ± SD for each group (*n* = 6). **P < 0.01 vs. the control group. ^#^*P* < 0.05 and ^##^*P* < 0.01 vs. the LPS-treated group.

We measured the levels of NLRP3 and caspase-1 p20 to estimate the effects of DHM on NLRP3 inflammasome activation. Compared to the LPS treatment, the 0.025% DHM treatment markedly decreased (*P* < 0.05) the protein expression levels of NLRP3 (Figure 9B) and caspase-1 p20 (Figure 9C), and the 0.05% and 0.1% DHM treatments significantly decreased (*P* < 0.01) their protein expression levels. These results revealed that the activation of the NLRP3 inflammasome was repressed by the administration of DHM. Compared with LPS treatment, treatment with 0.025%, 0.05% or 0.1% DHM significantly decreased (*P* < 0.01) the mRNA expression levels of NLRP3 (Figure 9D) and pro-caspase-1 (Figure 9E). These data indicated that the NLRP3 inflammasome was inhibited by DHM treatment.

### DHM inhibits pyroptosis in LPS-induced hepatic injury

**Figure 10:**
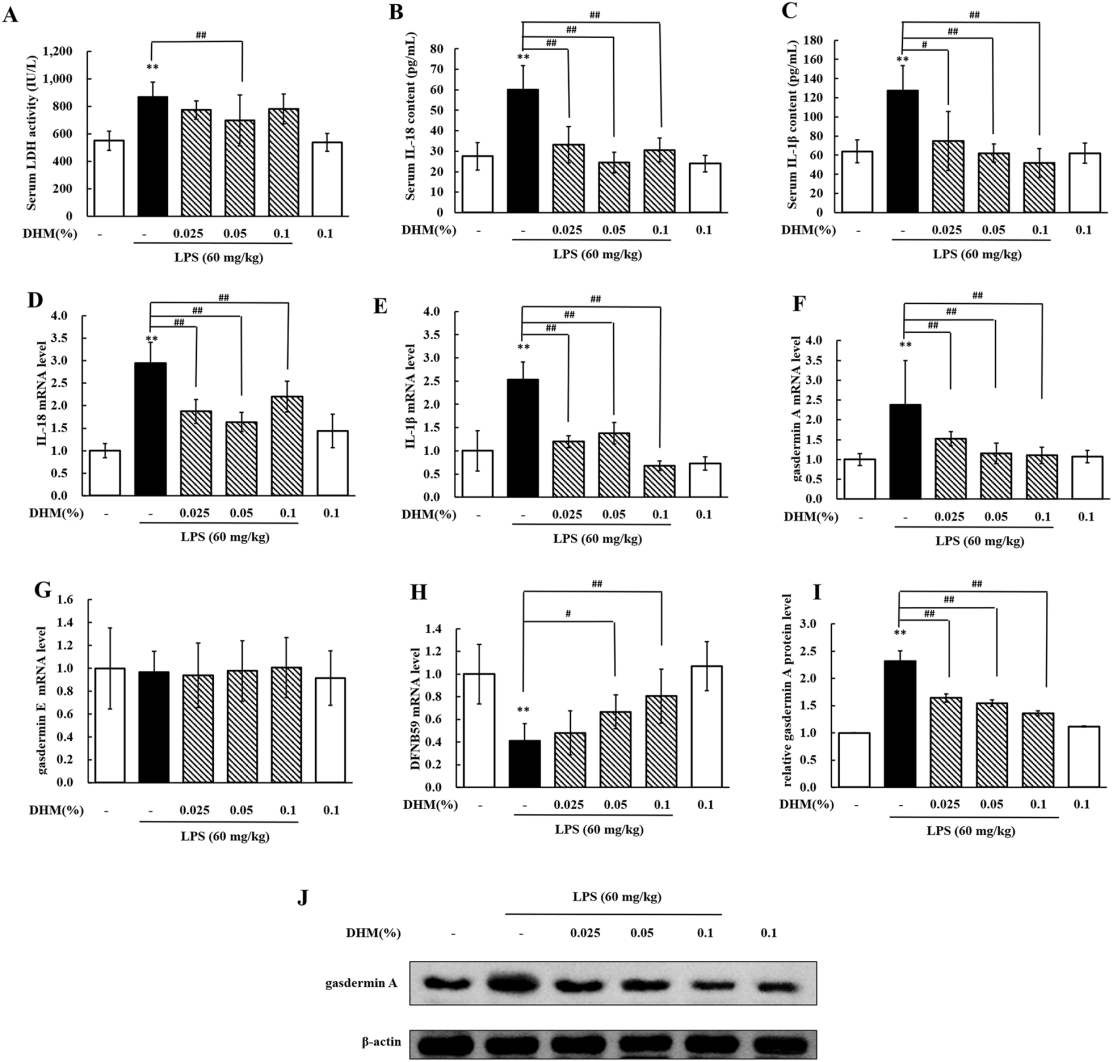
**DHM repressed pyroptosis in LPS-induced hepatotoxicity.** **A**–**C** Changes in LDH activity, IL-18 content and IL-1β content in serum from chickens administered 0.025%, 0.05% or 0.1% DHM for 14 days followed by LPS exposure (60 mg/kg). **D**–**H** Changes in the mRNA expression levels of IL-18 (fold change in IL-18/β-actin), IL-1β (fold change in IL-1β/β-actin), gasdermin A (fold change in gasdermin A/β-actin), gasdermin E (fold change in gasdermin E/β-actin), and DFNB59 (fold change in DFNB59/β-actin) in livers from chickens administered 0.025%, 0.05% or 0.1% DHM for 14 days followed by LPS exposure (60 mg/kg). **I** Changes in the protein expression level of gasdermin A (fold change in gasdermin A/β-actin) in the livers of chickens administered 0.025%, 0.05% or 0.1% DHM for 14 days followed by LPS exposure (60 mg/kg). **J** Western blot analyses of for the expression of gasdermin A and β-actin (loading control) in total tissue lysate. Values are expressed as the mean ± SD for each group (*n* = 6). **P < 0.01 vs. the control group. ^#^*P* < 0.05 and ^##^*P* < 0.01 vs. the LPS-treated group.

We further evaluated the effects of DHM on pyroptosis in LPS-induced hepatic injury. The serum levels of LDH, IL-1β, and IL-18 and the liver mRNA expression levels of IL-1β and IL-18 were significantly increased (*P* < 0.01) in the LPS group compared with the control group (Figures 10A–E). Treatment with DHM reduced serum LDH activity; this decrease was significant (*P* < 0.01) in the 0.05% DHM group compared to the LPS group (Figure 10A). Compared with the LPS treatment, the 0.025%, 0.05% and 0.1% DHM treatments significantly decreased (*P* < 0.01) IL-1β and IL-18 contents in serum (Figures 10B and C) and IL-18 and IL-1β mRNA expression levels in the liver (Figures 10D and E). Treatment with 0.1% DHM alone did not change LDH, IL-1β or IL-18 levels. These results revealed that DHM could alleviate pyroptosis in LPS-induced hepatic injury. In addition, the expression levels of the three paralogous genes in the gasdermin family in chickens, *gasdermin A*, *gasdermin E* and *DFNB59*, were evaluated. The mRNA expression level of gasdermin A was significantly increased (*P* < 0.01, Figure 10F) in LPS-induced hepatic injury compared with the control condition. The LPS treatment-induced increase in the mRNA expression level of gasdermin A was significantly decreased (*P* < 0.01, Figure 10F) by DHM treatment in a dose-dependent manner. As shown in Figure 9G, the mRNA expression level of gasdermin E did not change with the administration of LPS or DHM. However, the mRNA expression level of DFNB59 was significantly decreased (*P* < 0.01) in the LPS group compared with the control group (Figure 10H). DHM treatment increased the mRNA expression level of DFNB59 in a dose-dependent manner. In addition, we detected the protein expression level of gasdermin A to further evaluate the effects of DHM on pyroptosis. As shown in Figures 10I and J, the protein expression level of gasdermin A was significantly increased (*P* < 0.01) in chickens treated with LPS compared with nontreated chickens, while compared with LPS treatment, cotreatment with DHM and LPS significantly decreased (*P* < 0.01) the expression level of gasdermin A. These data indicated that DHM inhibited pyroptosis in LPS-induced liver damage.

## Discussion

*E. coli* has caused very large economic losses to the poultry industry. LPS, the main pathogenic factor and major component of the outer membranes of *E. coli*, is released after the death or division of bacteria, especially with antibiotic treatment [24]. Resident macrophages, Kupffer cells, are responsible for removing LPS from blood [25]. However, a large dose of LPS can deplete Kupffer cells and subsequently cause severe hepatic injury. Intraperitoneal injection with 10–30 mg/kg LPS can cause liver injury in mice [3, 26], and injection with 50–200 mg/kg LPS causes liver injury in chickens [4, 27]. In the present study, the serum levels of AST, ALT and DBILI increased after 60 mg/kg LPS treatment. In addition, treatment with LPS caused severe pathological changes as evidenced by histopathological and ultrastructural analyses. Our data suggested that intraperitoneal injection of 60 mg/kg LPS can cause hepatic injury in chickens.

The available evidence shows the important role of the NLRP3 inflammasome in liver damage. Chen et al. reported that the protein expression levels of NLRP3 and pyroptosis-related proteins increased in caecal ligation- and puncture-induced liver injury, but liver damage and mortality were suppressed by inhibiting the NLRP3 inflammasome [28]. A recent study demonstrated that hepatic NLRP3 was activated in Schistosoma-induced liver injury in mice, but NLRP3^−/−^ mice were protected from Schistosoma-induced liver injury [29]. In humans and mice, the NLRP3 inflammasome is a complex containing the receptor protein NLRP3, the adaptor protein apoptosis-associated speck-like protein containing a CARD (ASC) and the effector protein pro-caspase-1; however, ASC does not exist in chickens [30]. Thus, we measured the expression of NLRP3 and caspase-1 to evaluate the activation of the NLRP3 inflammasome in the present study. The increased expression levels of NLRP3, pro-caspase-1 and caspase-1 p20 revealed that the NLRP3 inflammasome was activated when chickens were treated with LPS. Chickens subjected to LPS-induced hepatic injury showed increased serum levels of IL-1β, IL-18 and LDH, indicating that pyroptosis was involved in LPS-induced hepatic injury. Notably, gene knockout or NLRP3^−/−^ chickens were not available at the time of this study, so an NLRP3 inhibitor was used in this study. MCC950 is a highly selective inhibitor of the NLRP3 inflammasome, and it can block NLRP3 inflammasome activation with no effect on other inflammasomes [19]. It was demonstrated that MCC950 could alleviate cholestatic liver injury in a bile duct ligation model in mice [31]. Our results showed that the NLRP3 inflammasome was blocked in chickens cotreated with MCC950 and LPS compared with those treated with LPS alone. In addition, pyroptosis occurred in LPS-induced hepatic injury, and it was reduced after blocking the NLRP3 inflammasome. Decreased serum ALT and AST activities and histopathological analyses suggested that the inhibition of NLRP3 alleviated LPS-induced hepatic injury. Our data demonstrated that the NLRP3 inflammasome and pyroptosis were involved in *E. coli* LPS-induced hepatic injury in chickens.

DHM is one of the flavonoids extracted from *Ampelopsis grossedentata*, comprising more than 30% of the dry weight of vine tea (the tender stems and leaves of *Ampelopsis grossedentata*) [32]. DHM-rich herbal mixture extract (0.8% in daily diet in rats) is considered a nontoxic herbal prescription and can be used as a functional food, food additive and natural remedy [13]. In this study, we investigated the protective effect of DHM against LPS-induced hepatic injury in chickens. Serum biochemical analyses showed that pretreatment with 0.05% DHM alleviated the hepatic injury caused by LPS. Our histopathological and ultrastructural imaging results demonstrated that DHM showed a potent protective effect against hepatic injury in a dose-dependent manner. DHM (0.05% mass fraction) is approximately 100 mg/kg according to body weight and feed intake. It was reported that when mice were given DHM at a range of concentrations from 150 mg/kg to 1.5 g/kg, there were no significant side effects [33]. In the present study, treatment with 0.1% DHM alone for 14 days did not have any effect on liver function or histopathological and ultrastructural observations in chickens, showing that DHM is a nontoxic food supplement. However, the acute toxicity and long-term toxicity of DHM in chickens still need to be assessed.

Oxidative stress, an imbalance between oxidant and antioxidant agents, is a phenomenon that accelerates liver injury progression [34]. Oxidative stress is characterized by the depletion of antioxidant proteins and the accumulation of superoxide. It was reported that DHM increased the activities of SOD and GSH-Px in a diabetes mouse model [35]. We assessed oxidative stress-related parameters to evaluate the effects of DHM on oxidative stress in LPS-induced hepatic injury. The increased levels of MDA and H_2_O_2_, along with the decreased activities of SOD and GSH-Px, in the LPS-treated group revealed that oxidative homeostasis was destroyed in liver inflammatory injury. Our data suggested that DHM could improve hepatic oxidative homeostasis in a dose-dependent manner by enhancing antioxidant capacity and repressing oxidative stress. Interestingly, treatment with 0.1% DHM for 14 d had no effect on oxidative homeostasis in control chickens.

Several studies have shown that traditional therapeutic herbs can affect the NLRP3 inflammasome. Pretreatment with *Cinnamomum osmophloeum* essential oil decreased the expression of ASC, caspase-1, and NLRP3 in the intestinal mucosa and the serum levels of IL-1β and IL-18 [36]. Another study demonstrated the protective effects of *Syneilesis palmata* extract against LPS-induced endotoxin and *E. coli*-induced sepsis mouse models via the inhibition of NLRP3 inflammasome activation [37]. Since the NLRP3 inflammasome participated in LPS-induced liver damage, we wondered whether DHM could affect the NLRP3 inflammasome. Here, 0.05% DHM treatment reduced the expression levels of NLRP3, pro-caspase-1 and caspase-1 p20 in liver tissues, demonstrating that DHM could reduce the activation of NLRP3 in LPS-induced hepatic injury. Since ROS are considered to activate the NLRP3 inflammasome, we speculated that ROS may be involved in the inhibitory effects of DHM on the NLRP3 inflammasome. Recently, one study reported that naringenin, a flavonoid compound, could downregulate NLRP3 to attenuate nonalcoholic fatty liver in mice [38]. In the present study, we demonstrated that DHM could inhibit the activation of the NLRP3 inflammasome to protect the liver against LPS stimulation. However, DHM did not have any effect on NLRP3 inflammasome activation in chickens that were not treated with LPS.

Pyroptosis is a form of programmed inflammatory cell death, and its involvement in nonalcoholic steatohepatitis in mice and humans has been demonstrated [39]. The important inflammatory cytokines involved in the development of liver injury, IL-1β and IL-18, are the main products of pyroptosis. These two inflammatory cytokines can be released after pyroptosis, followed by the activation of other inflammatory cells and amplification of the inflammatory response [40]. In the present study, our data showed that administration of DHM decreased hepatic pyroptosis in liver injury. Gasdermins are executor proteins of pyroptosis, and there are six paralogous genes in humans; however, there are only three in birds [41]. Notably, *gasdermin A*, *gasdermin E* and *DFNB59* genes are found in vertebrates, while *gasdermin B*, *gasdermin C* and *gasdermin D* are exclusively present in the genomes of mammals [42]. We detected the mRNA expression levels of the three gasdermins to study the changes in gasdermin expression in hepatic damage. Our data showed that at the transcriptional level, gasdermin A was upregulated while DFNB59 was downregulated by LPS administration, but no effect on gasdermin E was observed. Interestingly, DHM treatment downregulated gasdermin A and upregulated DFNB59 in LPS-induced hepatic injury. Gasdermin A, gasdermin E and DFNB59 are three proteins that belong to the gasdermin family, but gasdermin E and DFNB59 also belong to the subclass of deafness-associated proteins [41]. In the present study, the changes in gasdermin E and DFNB59 levels were not consistent with the changes in IL-1β and IL-18 levels. Because of the inconsistent changes in gasdermin E and DFNB59 mRNA expression and the lack of available antibodies for chickens, we did not detect their protein levels. The mRNA and protein expression levels of gasdermin A indicated that gasdermin A might be the key protein involved in pyroptosis in LPS-induced hepatic injury in chickens and that DHM might have inhibitory effects on gasdermin A and pyroptosis. Our data demonstrated that DHM repressed pyroptosis during LPS-induced hepatic injury. However, the specific mechanism of gasdermin A in pyroptosis in chickens still needs further study.

Based on the data presented above, this study demonstrated that the NLRP3 inflammasome is an important mechanism in *E. coli* LPS-induced hepatic injury. The NLRP3 inflammasome was activated in LPS-induced hepatic injury, while inhibition of NLRP3 ameliorated LPS-induced hepatic injury. This is the first report indicating that DHM could alleviate LPS-induced hepatic injury, and the mechanisms of its protective effects might involve inhibition of oxidative stress, the NLRP3 inflammasome and pyroptosis. These findings improve our understanding of the mechanism of LPS-induced hepatic injury and the protective mechanism of DHM and provide a reference for the future development of therapeutic strategies.

## Data Availability

The datasets used and/or analysed during the current study are available from the corresponding author upon reasonable request.

## Abbreviations

ALT: alanine aminotransferase
APEC: avian pathogenic *Escherichia coli*
AST: aspartate aminotransferase
DBILI: direct bilirubin
DHM: dihydromyricetin
*E. coli*: *Escherichia coli*
ELISA: enzyme-linked immunosorbent assay
GSH-Px: glutathione peroxidase
IL-1β: interleukin 1β
IL-18: interleukin 18
LDH: lactate dehydrogenase
LPS: lipopolysaccharide
MDA: malondialdehyde
NLRP3: nucleotide-binding and oligomerization domain (NOD)-like receptor pyrin domain-containing 3
caspase1: cysteinyl aspartate specific proteinase 1
SOD: superoxide dismutase
